# Characteristics in the Population of Stray Dogs and Changes After One Year From a City in Southern Mexico

**DOI:** 10.1155/vmi/5479606

**Published:** 2025-06-02

**Authors:** A. Novelo-Sanguino, M. Jiménez-Coello, J. C. Segura-Correa, A. Ortega-Pacheco

**Affiliations:** ^1^Department of Animal Health and Preventive Medicine, Faculty of Veterinary Medicine, Autonomous University of Yucatan, Mérida, Yucatán, Mexico; ^2^Biomedical Unit, Regional Research Center “Dr. Hideyo Noguchi”, Autonomous University of Yucatan, Mérida, Yucatán, Mexico

## Abstract

The present study generated evidence on the population of stray dogs in the city of Mérida Yucatán, Mexico. The sighting method was used using the “count within a selected block” method recommended by the World Society for the Protection of Animals. For the sample size, 21 blocks were randomly selected from the city. The calculation of the density of the dogs was obtained considering the extension of 186.24 km^2^ of the city, and the characteristics of the dogs were evaluated through direct observation. The same protocol was performed 1 year later. The estimated number of dogs was 4764 ± 478 in 2022 and later increased to 7650 ± 779 in 2023 (*p* < 0.006). The density of the dog population in 2022 was 25.6 ± 2.6 animals/km^2^, with a dog-human ratio of 0.5:100. In 2023, a population density of 41.1 ± 4.3 animals/km^2^ and a dog-human ratio of 0.8:100 were obtained. This significant increase may be due to more food availability and capacity to achieve a successful reproduction in the first year of observation. However, bias may be considered when using any counting methodology of stray dogs since their mobility may be intensely variable. The male-female ratio was 2.4:1 for 2022 and 2.3:1 for the year 2023. Most of the dogs were adults, of mixed breed, and with solitary behaviour in both years. The body condition in 2022 was predominantly ideal, with a significant decrease in 2023 (*p* < 0.001) probably because of the increased number and competence for food when the second evaluation was performed. When comparing the population according to the areas of the city, the southern area had a higher proportion of thin dogs in both years, with an increase of up to 30% (*p* < 0.001) by 2023. The presence of dermatological conditions in the dogs increased from 4% in 2022 to 15% in 2023 (*p* < 0.001). It is concluded that the number and characteristics of stray dogs studied presented changes over a year time probably because of the carrying capacity of the environment, with differences between areas of the city, particularly in lower socioeconomical areas.

## 1. Introduction

A stray dog is that animal that is not under the direct control of a person or that is not prevented from roaming freely [1]. The increase in stray dog populations is currently a public and animal health problem. It is estimated that there are close to 700 million stray dogs in the world, of which more than 60% appear to have or have had an owner [2]. In Latin America, figures vary; for example, in a city in Chile, 64,794 street dogs have been registered [3], 726 in Ecuador [4] and 1411 ± 643 in a city in Peru [5]. In Mexico, in 2018, it was reported about 16.1 million stray dogs [6].

As a consequence of a high number of stray dogs, particularly in developing countries such as the studied area, the risk of contracting zoonotic diseases is high. For instance, the environmental contamination with fresh faecal matter in streets may favour the spread of zoonotic diseases (mainly parasitic agents such as *Toxocara* spp. and *Ancylostoma duodenale*), particularly in children playing in sand parks with direct contact of fresh faeces [7–10]. It has also been documented for instance in India, that dog bites represent a high proportion of all bites caused to humans [11]. It has been highlighted that about 60% of bites are implied by stray dogs [12]. In the city of Mérida, Yucatán, a total of 2450 cases of dog-to-human bites were reported in 2020, with 40% of the reports caused by stray dogs (Animal Control Centre, Merida, Personal communication, November 15, 2021).

In addition, stray dogs have a direct effect on human health, since there are more than 65 zoonotic diseases (the majority dangerous for humans) such as rabies, leptospirosis, hookworm, larva migrans, ehrlichiosis, brucellosis, cestodiasis, salmonellosis and echinococcosis, among others, which are usually spread by stray dogs accessing public areas such as gardens and parks where the child population may be more predispose to contagion [13, 14]. Fortunately, in 2019, the World Health Organization validated Mexico as the first country to eliminate rabies transmitted by dogs as a public health problem [15].

The population dynamics of stray dogs allows us to know the composition and characterization of the population, such as age, the number of individuals, the female-male ratio, and the body condition of the individual dog [16]. The comparisons of these indicators provide an overview of the number of the dog population, whether there is an increase or decrease over time, and whether there are changes in their conformation [17]. Therefore, understanding the fluctuation in numbers and characterization of stray dogs is essential for developing programs that reduce the impact caused by these populations on the environment, public health [2, 16, 17] and animal welfare.

Since the population growth of dogs is a dynamic process, and to our knowledge, most of the published studies on the stray dog population are performed on a single point of time during the year without further evaluation, the present study had the objective of comparing the yearly changes in the structure and the population of stray dogs in Mérida, capital city of Yucatán, México.

## 2. Materials and Methods

### 2.1. Study Design

The present study is a descriptive, comparative cross-sectional type carried out on stray dogs in the City of Mérida, Yucatán, Mexico. Because of the nature of the study, no ethical approval was required.

### 2.2. Study Area

The study was conducted in the city of Merida, Yucatan, Mexico (20°41′49.56″ to 21°11′27.96″ North latitude and 89°48′04.32″ to 89°27′04.68″ West longitude). The city of Mérida has an area of 186.24 km^2^ and a population of approximately 995,000 inhabitants reported in 2020. The climate of this region is warm and subhumid with summer rains. It has an annual temperature of 19.2°–33.5°C with an average of 26.4°C. The average relative humidity is 80%, and an annual rainfall is of 1200 mm [18].

### 2.3. Stray Dog Number Estimate

Stray dogs' population was estimated by sighting, using the “count within a selected block” method [17]. Based on this methodology, the city was divided into four subregions (northeast, northwest, southeast and southwest). The subregions were divided into 30 blocks (0.30 km^2^), so that each block could be covered on foot in less than 2 hours. For the selection of the sample of blocks, a colour was assigned to each block (red, purple, brown and green), without repeating colour between contiguous blocks, and the same number of blocks per colour was obtained. Thus, each block had a 25% probability of being selected. One of the four colours was randomly drawn, and all blocks assigned to the colour were selected. Each of the selected blocks was numbered in the reading direction, that is, from left to right. Twenty-one blocks were randomly selected. There was selected a total of 12 blocks in the northern zone and 9 blocks in the southern zone. The total number of dogs for the region was found to be between the limits of two standard deviations. This methodology allows a good covering of the whole city. The stray dog number estimation was calculated using the following formula:

(1) $$\begin{array}{c} \text{Stray dog number}=\frac{\text{Total number of dogs counted}}{\left(\left(\text{Number of blocks selected}\right)/\left(\text{Total number of blocks}\right)\right)}. \end{array}$$

### 2.4. Stray Dogs' Population Density

The density of the stray dog population was obtained from the number of stray dogs counted in 186.4 km^2^ in the city of Mérida, considering the delimitation of the peripheral city borders and the subdivisions contiguous to the peripheral borders of the city. The dog-human relationship in both years was determined from the division between the number of inhabitants in the city reported in 2020 [18] (*n* = 995,129) and the estimate of stray dogs, and the result was expressed as the number of stray dogs per 100 humans.

### 2.5. Characterization of the Stray Dogs

For the characterization of dogs, the variables of sex (male/female), age (puppy/adult), race (pure/crossbreed/mixed), body condition (thin/ideal/overweight), visible dermatological conditions (absence/presence), visible injuries, lameness, presence of secretions or masses (absence/presence), behaviour (solitary/pack) and sexual interaction (absence/presence) were considered. The breed of dogs was determinate subjectively by their phenotypical appearance: A dog was considered purebred when its phenotypic appearance met the characteristic standards of a specific breed. A crossbred dog was considered when some purebred phenotypic characteristics could be identified, and a mongrel dog (“malix” meaning “ordinary” in Mayan language) was considered when there were no identifiable characteristics of a purebred dog [19].

### 2.6. Data Analysis

Statistical analysis was performed using the IBM SPSS Statistics 25 program. Quantitative variables (such as the number of animals) were evaluated using Shapiro–Wilk Z (95% CI, *p* < 0.05) to analyse the type of distribution. Qualitative variables such as sex, race, age and body condition were expressed as frequencies and percentages. For dogs in the north (northeast and northwest) and south zones of the city (southeast and southwest), a comparative analysis of the different variables evaluated (the total number of stray dogs, sex, breed, age, etc.) was performed using the Student's T statistic for related samples or Wilcoxon, Mann–Whitney U or Student's *T*-test for independent samples and chi^2^ or Fisher's exact test (95% CI, *p* < 0.05) depending on the characteristics of the variables.

## 3. Results

### 3.1. Estimation of Stray Dog's Number

During the survey period (February–April) of 2022 and 2023, the same 21 city blocks were covered. The estimated stray dog's number was 4764 ± 478 and 7650 ± 779 (95% CI) for the years 2022 and 2023, respectively. When comparing the estimates, a greater number of animals was found in the year 2023 (*p* < 0.006). No differences were found between years in the stray dog estimates for the northern and southern areas of the city (Table 1). When comparing between zones (north vs. south), a greater number of stray dogs was found in the southern zone in both years.

### 3.2. Stray Dogs' Density

The density of the stray dogs in 2022 was 25.6 ± 2.6 dogs per km^2^, with a dog-human ratio of 0.5:100. In 2023, a population density of 41.1 ± 4.3 dogs per km^2^ and a dog-human ratio of 0.8:100 were obtained.

### 3.3. Characteristics of the Stray Dogs

Sex of the total stray dogs was observed in the 2022 survey: 71% of the population were males, and 29% were females. The male-female ratio was 2.4:1 in 2022; similar values were obtained in 2023, where 70% of dogs were males and 30% were females; the male-female ratio was 2.3:1 (Tables 2 and 3).

Breed: The mongrel was the most frequent dog, representing 85% and 83% of the stray dog population in the estimates of 2022 and 2023, respectively; there were no differences in the proportion of breeds between the northern and southern areas for 2022. However, a significant number of purebred and crossbreed dogs was recorded in the northern zone for 2023 (Tables 2 and 3). Similarly, the proportion of mongrel dogs increased from 8% in 2022 to 24% in 2023 in the northern zone.

Age: The majority of observed stray dogs were adults in both years. However, the proportion of adult increased from 88% in 2022 to 96% of the population in 2023. A higher proportion of pups (15%) was observed in the southern zone compared to the northern zone (3%) in 2022 (Tables 2, 3, 4), and the proportion of pups in the southern zone in 2022 (15%) was even higher than the estimated proportion of pups (5%) for the same zone in 2023 (Tables 2 and 3).

Body condition: The most frequently recorded body condition in 2022 was ideal in 63% of the evaluated population. By 2023, the thin body condition was 69%, which represents a 35% increase compared to 2022 (Table 4). When comparing zones, the southern zone observed the highest proportion of thin dogs in both years, with a value of 20% in 2022 and a later increase of 30% in the same zone in 2023 (Tables 2 and 3). On the other hand, when comparing the zones between years, it was observed that the northern zone showed an increased in the proportion of dogs with a thin body condition from 20% in 2022 to 45% in 2023. Similarly, in the southern zone, an increase in the proportion of dogs with a thin body condition was observed from 40% in 2022 to 75% in 2023 (Tables 2 and 3).

Dermatological condition and injuries: The presence of visible alopecic areas in the integument of the dogs was considered as a dermatological condition. In 2022, the presence of dermatological conditions in the canine population was 4%; by 2023, this proportion increases up to 15% (Table 4). Similar results were found in the southern zone for 2022 and 2023, whose values were 5% and 16%, respectively. The presence of lameness, masses, or discharge detected with the naked eye in the canine population observed was considered as a lesion. The proportion of injuries in the dogs was 6% for both years, and no significant differences were found when comparing the proportions between areas and years evaluated (Tables 2, 3, 4).

Behaviour and sexual interaction: The solitary activity or pack activity (presence of > 2 individuals) was determined in the stray dogs at the time of observation and was considered as part of a behaviour. Most of the observed dogs displayed solitary behaviour in both years, being 53% and 58% of the population for 2022 and 2023, respectively (Table 4). In 2023, a higher proportion of dogs in packs was observed in the southern zone compared to the northern zone (46% vs. 27%) (Table 3). When comparing zones between years, it was observed that the pack behaviour reduced by 20% in 2023 compared to 2022 for the northern zone, whilst in the southern zone no significant changes were found between years. The sexual interaction of dogs was also evaluated considering any event related to copulation or attempted copulation between dogs of opposite sex. In 2022, the presence of sexual interaction in the dogs was 4%, with no significant differences between areas found. In 2023, the presence of sexual interaction was observed in 5% of the dogs with a difference of 6% between zones (Tables 2 and 3). Regarding the comparison by zones between years, the northern zone had a 6% decrease in the population of dogs with sexual interaction in 2023 compared to 2022. In the southern zone, there were no significant changes in the comparison between years.

## 4. Discussion

The estimation and characterization of the stray dog population and its dynamic is not an easy task due to the constant growth of the city, movement of animals, environmental factors, incoming new animals to the city and the lack of legislation on the mandatory registration of pets. Management of stray dog populations represents a challenge for many countries around the world. Inadequate control of the stray dog population represents a problem for public health, as well as animal welfare. Therefore, knowing the dynamics of their population will allow the elaboration of more efficient and specific control programs adequate to every region to obtain successful results. To the best of our knowledge, this study is the first to estimate and characterize the stray dog in the city of Mérida, Yucatán, and the third performed in Mexico [9, 10], and it uses for the first time a yearly follow-up monitoring.

Diverse methods have been used to estimate the size of stray dog populations. In the present study, the counting method within a block was used [17]. Count-based methods have been used in studies of stray dog estimates in countries such as Peru [5], Bangladesh [20, 21], Chile [3], Russia [22], Iran [23] and Nepal [24]. Other methods to estimate roaming dog populations include photographic capture and recapture used in countries such as Brazil [25], India [26] and Mexico [9]. Methods based on sightings, such as photography and the application of surveys, are characterized by being less invasive as they do not have contact with the animals, which reduces the probability that researchers are attacked and exposed to zoonotic diseases, in addition to being less expensive than methods that require capture and marking [16, 23]. One of the limitations of the photographic method is that it is not always possible to obtain high-quality photographs that guarantee the identification of the characteristics of the animals sighted, especially when a pack of dogs is photographed [23]. However, the methodology applied in this study allowed detailed observation of the characteristics evaluated in the stray dog population.

The present study estimated yearly increase in the stray dog numbers from 4764 ± 478 in 2022 to 7650 ± 779 in 2023. Studies on stray dog populations around the world have reported different estimates but include only one single observation; similar numbers have been reported in Iran (6781 stray dogs) [23] and Afghanistan (1821 ± 256 stray dogs) [27], but in other countries such as Kathmandu, the population of stray dogs may be much higher (22,286 ± 3067 stray dogs) [24]. In Latin American countries, populations of stray dogs also varies; for instance, in Peru, an estimate of 1411 ± 643 stray dogs is observed during the day and 922 ± 497 at night [5]; in Chile, 214,933 stray dogs are reported from the streets [3]; in Mexico, a range of 63,000 to 80,000 stray dogs was estimated in the south of the country [9], whereas in the Caribbean, Tulum, 310 free roaming dogs were estimated [10]. Clearly, the great variation in the number of stray dogs varies depending on the methodology used to estimate the number of dogs and its repetitions and most of all the environmental carrying capacity to maintain dogs of each region and the specific policies and actions taken for their control. As is known, population dynamics changes in the conformation over time [16]. In the present study, the number of counted dogs increased by 60% in 1 year. However, this cannot be extrapolated to the whole population of stray dogs in the city. Several factors may have influence this apparent growth in the number: dog moving to different areas, food availability, climatological conditions and human activities may have influenced these results. However, the information here showed demonstrate that several surveys are necessary to obtain more robust and accurate data for the planning of effective programs aiming to their control and improving of welfare. In Mérida, there are active programs aimed at the management and control of dogs (sterilization campaigns, laws for animal abuse and a municipal dog shelter), but since no evaluations have been performed, the impact on the stray dog population is unknown. However, the carrying capacity of the environment may limit its growth to reach high dog density as in places where programs to control dog populations are absent; in this case, the stray dog population may be reaching the maximum environmental carrying capacity; for this reason, several evaluations may be necessary to evaluate the population of stray dogs in a place. At least yearly evaluations during several years may give a more realistic view of the dynamic; dog populations can grow as human populations' growth [28].

A study of socio-spatial segregation in Mérida identified that the upper and middle upper socioeconomic class is in the central and northern part of the city, while the lower and lower middle socioeconomic class is located mostly in the southern part and periphery of the city [29]. This could explain the higher proportion of stray dogs observed in the southern zone of the city, since it has been claimed that low/medium socioeconomic class neighbourhoods feed stray dogs more frequently [30], which leads to a greater agglomeration of stray dogs near food sources [31], thus increasing the carrying capacity of the environment. However, cultural differences may account from distinctive countries in the world where stray dogs are commonly found in the cities.

The stray dog population density found in this study is around those reported by various communities around the world such as in a province of Iran (30.8 stray dogs/km^2^) [23] in two cities of Bangladesh with 14 stray dogs/km^2^ [20] and 52 stray dogs/km^2^ [21] and Tulum, Mexico (48.57 stray dogs/km^2^) [10]. However, in the city of Campeche, Mexico, a density of 1081 stray dogs/km^2^ is reported, a figure that is above the estimate found in this study and many countries of the world [9]. This difference may be associated with the lack of stray dog control programs, food abundance and the fact that at the time of the study, Campeche does not have a domestic fauna management centre [9].

Despite the fact that the density of stray dogs is a reference to the size of the populations, the dog-human relationship index is a better indicator when creating management programs and evaluating the growth of stray dog populations [23, 32]. The stray dog-human ratio obtained in this study (0.5:100 in 2022 and 0.8:100 in 2023) is similar to 0.25:100 reported in Turkey [33] and 0.83:100 in Bangladesh [20] but lower than 1.2:100 reported in Iran [23], 2.6–3.6:100 and 2.4–3.1:100 in rural and urban areas of India [34] and in Campeche, Mexico (43:100) [9]. The importance of this ratio is that when the dog-human ratio is 10:1 or higher, the risk of zoonotic diseases is increased [35].

In the present study, a higher proportion of males was found in both years. Similar ratios have been reported in Mexico (2.5:1) [9], India (2.4:1) [36] and Bangladesh [20] (2:1); a higher proportion of males (3.2:1) is also reported in Iran [23] South Africa (1.8:1) [37] and Afghanistan (1.6:1) [27] but in a lower proportion compared with the present study. An investigation carried out [38] refers that the citizens of Merida Yucatan in Mexico have a slight preference for female dogs and therefore relinquish males in the streets. However, studies are needed to corroborate the above. Other studies attribute the greater presence of male stray dogs due to the higher mortality occurring in females [36, 37]. However, preferences of dog sex may vary; in Dominica, male dogs are kept, allowing around 30% of them to freely roam [39].

It was found that mongrels were predominant in both years and that the pure breed and crosses increased by the year 2023 in the northern zone. Similarly in Bali [40], dogs were classified with and without owners into three breeds, the mixed breed (mongrel) being the most frequent (45.2%). A study carried out on dogs residing in Mérida found the mongrels (“malix”) as the most common in homes (37.4%) [19]. Similarly, considering the socio-spatial segregation of Mérida described [29], the increase in purebreds in the northern zone of the city could be due to the fact that families have a higher socioeconomic level and economical capacity to buy pure breed dogs and they are freely roaming probably because they escape from the houses and are not necessarily relinquished.

The high adult dog population estimated in this study is similar to findings reported in other countries with ranges of 77.5%–90.1% [23, 27, 34, 36]. Possibly the low proportion of puppies in the population studied is due to a high mortality rate in puppies < 3 months of age, or to human influence (vehicular accidents and intentional poisonings) [27, 36, 37], or due to the susceptibility of contracting diseases such as parvovirus and canine distemper [27]. In fact, litters being raised successfully away from human shelter are very rare, and the population growth occurs when recruiting individuals from the supervised dog population [41].

The ideal body condition was the most frequent in 2022, similar to that reported by other authors [9, 23, 27]. However, in 2023, this population presented changes more frequently with a thin body condition (*p* < 0.001). These findings may be due to the carrying capacity of the city, limiting the availability of food, and increasing competition for it. Although lower socioeconomic class neighbourhoods feed stray dogs more frequently [30], the increase in the population in southern Merida probably leads to greater competition and therefore less food availability, and together with low food quality, may be involved in the body condition of dogs.

In the present study, a significant higher proportion of stray dogs with dermatological conditions was observed in the 2023, with a significant increase in the southern zone of the city. These findings are similar with the skin lesions registered in stray dogs and unowned dogs, which range between 7.7% and 26% [9, 27, 39]. In Merida, skin lesions in stray dogs are highly associated with skin mites such as *Demodex canis* (23%), *Sarcoptes scabiei* var. *canis* (7%) and *Otodectes cynotis* (3.5%) [42]. Therefore, these agents could be the cause of the dermatological conditions observed in the study. The proportion of injuries in the stray dogs was 6% for both years, similarly to other studies [9] reporting visible lesions in 3.7% of the stray dog. Although the causes of the injuries were not determined, they could be attributed to car accidents or interactions with humans.

A study carried out in India on the daily activity time of stray dogs found that 47.7% of their time contributes to individual activities (walking and grooming, among others), while close to 14% of their total daily time corresponds to activities with peers or with humans [12]. These results are similar to what was observed in the present study, where solitary behaviour was the most frequent in both years; as known, dogs lost most of their pack behaviour after its domestication and solitary behaviour is expected.

Knowing the frequency of sexual interaction in stray dogs could be a good indicator to evaluate their potential growth; it is described that mating related behaviour in India may occur during the year at any time of the day [43], similarly, as described in Mérida [19] and Bahamas [44]. Thus, the capacity of stray dogs to reproduce all year round should be considered when studying dynamics on its population and when designing control strategies to control its reproduction.

## 5. Conclusions

This study demonstrates that one single measurement is not enough to assess the population dynamics of stray dogs, since in 1 year, there can be an important variation in the number of stray dogs. Changes are consequence of the capacity of the environment to reduce or promote reproduction and indicate probably bias because of the mobility of dogs during the counting period. Results also revealed that when the population of stray dogs increases, it can generate negative changes in their health, particularly in lower socioeconomical areas of the city where more density of stray dogs is expected.

## Figures and Tables

**Table 1:** Yearly comparison of stray dogs' number in the city of Merida.

	Year		*p* value
	2022	2023	
Total	4764 ± 478	7650 ± 779	0.006^a^
North	1288 ± 240	1659 ± 218	0.261^a^
South	3626 ± 581	6278 ± 973	0.109^b^

*Note:* Data are presented as estimate ± 2 standard deviation. A statistical analysis was performed using the Wilcoxon test for related samples. Different letters (a, b) indicate statistical significance (*p* < 0.05).

^b^Statistical analysis was performed using the Student's *T*-test for related samples. Significance is considered with a value of *p* < 0.05.

**Table 2:** Characteristics of the stray dog population in 2022.

Variable	Total *n* (%)	North *n* (%)	South *n* (%)	*p* value
Sex				
Male	148 (71)	42 (71)	106 (71)	> 0.09^a^
Female	60 (29)	17 (29)	43 (29)	
Breed				
Pure	7 (3)	4 (7)	3 (2)	0.197^b^
Crossbreed	26 (12)	5 (8)	19 (13)	0.602^a^
Mongrel	177 (85)	50 (85)	127 (85)	--Ref.--
Age				
Adult	184 (88)	57 (97)	127 (85)	0.027^a^
Young	24 (12)	2 (3)	22 (15)	
Body condition				
Thin	72 (35)	12 (20)	60 (40)	0.009^a^
Ideal	132 (63)	46 (78)	86 (58)	--Ref.--
Overweight	4 (2)	1 (2)	3 (2)	> 0.09^b^
Dermatological lesion				
Present	9 (4)	2 (3)	7 (5)	> 0.09^b^
Absent	199 (96)	57 (97)	142 (95)	
Lesions				
L., T., S.	12 (6)	2 (3)	10 (7)	0.515^b^
N/L	196 (94)	57 (97)	139 (93)	
Behaviour				
Alone	110 (53)	31 (53)	79 (53)	0.950^a^
Pack	98 (47)	28 (47)	70 (47)	
Sexual interaction				
Presence	8 (4)	4 (6)	4 (3)	0.226^b^
Absent	200 (96)	55 (94)	145 (97)	

*Note:* Ref.: reference value.

Abbreviations: L., T., S = lameness, tumour, secretion; N/L = no lesion.

^a^Statistical analysis was performed using Chi^2^.

^b^Statistical analysis was performed using Fisher's exact test. Significance is considered with a value of *p* < 0.05.

**Table 3:** Characteristics of the stray dog population in 2023.

Variable	Total	North	South	*p* value
	*n* (%)	*n* (%)	*n* (%)	
Sex				
Male	234 (70)	56 (74)	178 (69)	0.478^a^
Female	100 (30)	20 (26)	80 (31)	
Breed				
Pure	10 (3)	5 (7)	5 (2)	0.032^b^
Crossbreed	48 (14)	18 (24)	30 (12)	0.008^a^
Mongrel	276 (83)	53 (69)	223 (86)	--Ref.--
Age				
Adult	319 (96)	73 (96)	246 (95)	0.68^a^
Young	15 (4)	3 (4)	12 (5)	
Body condition				
Thin	229 (69)	34 (45)	195 (75)	> 0.001^a^
Ideal	98 (29)	40 (52)	58 (23)	--Ref.--
Overweight	7 (2)	2 (3)	5 (2)	0.699^b^
Dermatological lesion				
Present	51 (15)	10 (13)	41 (16)	0.688^a^
Absent	283 (85)	66 (87)	217 (84)	
Lesions				
L., T., S.	18 (6)	5 (7)	13 (5)	0.570^b^
N/L	316 (94)	71 (93)	245 (95)	
Behaviour				
Alone	194 (58)	56 (73)	138 (54)	0.002^a^
Pack	140 (42)	20 (27)	120 (46)	
Sexual interaction				
Presence	16 (5)	0 (0)	16 (6)	0.27^b^
Absent	318 (95)	76 (100)	242 (94)	

*Note:* Ref.: reference value.

Abbreviations: L., T., S = lameness, tumour, secretion; N/L = no lesion.

^a^Statistical analysis was performed using Chi^2^.

^b^Statistical analysis was performed using Fisher's exact test. Significance is considered with a value of *p* < 0.05.

**Table 4:** Comparison of dog characteristics between years.

Variable	Year		*p* value
	2022	2023	
Sex			
Male	148 (71)	230 (70)	0.861^a^
Female	60 (29)	100 (30)	
Breed			
Pure	7 (3)	10 (3)	1.00^b^
Crossbreed	26 (12)	48 (14)	0.605^a^
Mongrel	177 (85)	276 (83)	--Ref.--
Age			
Adult	184 (88)	310 (96)	0.003^a^
Young	24 (12)	15 (4)	
Body condition			
Thin	72 (35)	229 (69)	0.001^a^
Ideal	132 (63)	98 (29)	--Ref.--
Overweight	4 (2)	7 (2)	0.217^b^
Dermatological lesion			
Present	9 (4)	51 (15)	0.001^a^
Absent	199 (96)	283 (85)	
Lesions			
L., T., S.	12 (6)	18 (6)	0.850^b^
N/L	196 (94)	316 (94)	
Behaviour			
Alone	110 (53)	194 (58)	0.272^a^
Pack	98 (47)	140 (42)	
Sexual interaction			
Presence	8 (4)	16 (6)	0.672^b^
Absent	200 (96)	318 (95)	

*Note:* Ref.: reference value.

Abbreviations: L., T., S = lameness, tumour, secretion; N/L = no lesion.

^a^Statistical analysis was performed using Chi^2^.

^b^Statistical analysis was performed using Fisher's exact test. Significance is considered with a value of *p* < 0.05.

## Data Availability

The data that support the findings of this study are available on request to journal editors and researchers from the corresponding author. The data are not publicly available owing to in-depth interviews containing information that could compromise the privacy of research participants.
